# YTHDF2 alleviates cardiac hypertrophy via regulating Myh7 mRNA decoy

**DOI:** 10.1186/s13578-021-00649-7

**Published:** 2021-07-15

**Authors:** Hongfei Xu, Zhen Wang, Miao Chen, Wenting Zhao, Tingting Tao, Liang Ma, Yiming Ni, Weidong Li

**Affiliations:** 1 Department of Cardiovascular Surgery, School of Medicine, The First Affiliated Hospital of Zhejiang University, Number 79 Qingchun Road, Hangzhou, China; 2 Department of Cardiology, School of Medicine, The First Affiliated Hospital of Zhejiang University, Hangzhou, China

**Keywords:** Heart failure, Cardiac hypertrophy, N6-methyladenosine (m6A) modification, YT521-B homology domain family (YTHDF) proteins, Myh7 (beta-myosin heavy chain)

## Abstract

**Background:**

Pathological cardiac hypertrophy is a major contributor of heart failure (HF), which seriously threatens human’s health world widely. Deregulation of m6A RNA methylation, and m6A methyltransferases and de-methyltransferases have been demonstrated to act essential roles in cardiac hypertrophy and HF. Here, we studied the potential roles and its underlying mechanisms of m6A Reader YTHDF proteins in HF. In this study, we constructed HF mouse model by transverse aortic constriction surgery. Primary cardiomyocytes were isolated and stimulated with isoproterenol (ISO) or phenylephrine (PHE) to induce myocardial hypertrophy.

**Results:**

Through single-cell RNA-seq analysis, immunofluorescent staining, HE staining, Western blotting, and real time-PCR detections, we found that YTHDF2 mRNA and protein level, but not YTHDF1 or YTHDF3, was significantly increased during HF development. YTHDF2 overexpression could efficiently alleviate cardiac hypertrophy. Furthermore, through immunoprecipitation accompanied with mass spectrometry analysis, Gene Ontology (GO) analysis, and Kyoto Encyclopedia of Genes and Genomes (KEGG) pathway analysis, we found that ISO stimulation did not evidently affect YTHDF2-interacting proteins. However, ISO or PHE stimulation significantly increased YTHDF2 protein interacting with Myh7 (beta-myosin heavy chain) mRNA, an important cardiac hypertrophy marker, in an m6A-dependent manner. Knockdown of Myh7 or deletion of the YTH domain of YTHDF2 reversed the protective effects of YTHDF2 on cardiac hypertrophy. Finally, we found that ISO or PHE stimulation promoted YTHDF2 protein expression through enhancing Ythdf2 mRNA stability in an m6A-dependent manner in cardiomyocytes.

**Conclusions:**

Overall, our results indicate that the m6A Reader YTHDF2 suppresses cardiac hypertrophy via Myh7 mRNA decoy in an m6A-dependent manner. This study highlights the functional importance of YTHDF2-dependent cardiac m6A mRNA regulation during cardiac hypertrophy, and provides a novel mechanistic insight into the therapeutic mechanisms of YTHDF2.

**Supplementary Information:**

The online version contains supplementary material available at 10.1186/s13578-021-00649-7.

## Introduction

Heart failure (HF), characterized by reduced cardiac function and left ventricular dilatation, is one of the leading causes of death all over the world, and the 5‐year survival rate of patients with HF is only about 50% [1]. Pathological cardiac hypertrophy, generally triggered by pressure overload, is a critical risk factor of HF [2]. Cardiac hypertrophy is initially considered as an adaptive response to produce sufficient force to match an increase in wall tension or increased workload, but could eventually lead to HF [3]. So far, although great achievements have been made in the treatment for HF, there is still no effective drugs for completely preventing the eventual progression of the disease [4]. Thus, understanding the molecular events leading to HF is essential for supplying specific targets for novel drug research and development.

N6-methyladenosine (m6A) modification, the most abundant internal chemical modification in RNA, plays critical roles in regulating RNA processing, nuclear output, translation regulation, and RNA degradation [5, 6]. Generally, m6A modification in RNA is dynamically and reversibly regulated, and occurs within a highly-conserved consensus motif RRACH (R = G or A, H = A, C or U) [7]. m6A modification is catalyzed by the methyltransferase complex, including methyltransferase-like 3 (METTL3), methyltransferase-like 14 (METTLl4), Wilms tumor 1-associated protein (WTAP), protein virilizer homolog (VIRMA), E3 ubiquitin-protein ligase Hakai (HAKAI), zinc finger CCCH domain-containing protein 13 (ZC3H13), RNA-binding protein 15 (RBM15), and methyltransferase-like 16 (METTL16) [8–10], and is removed by demethylases, such as ALKB homolog 5 (ALKBH5) and fat mass and obesity-associated protein (FTO) [11]. m6A modified RNA was recognized by “Reader” proteins, such as YT521-B homology domain family (YTHDF) proteins, to realize different biological functions [12–14]. Recently studies report that alteration of m6A RNA methylation contributes to HF progression [15], and enhanced m6A methylation leads to compensated cardiac hypertrophy [16]. Besides, cardiac-specific FTO knockout mice exhibit a more severe reduction in ejection fraction and a higher degree of dilatation upon transverse aortic constriction (TAC) surgery [15]. These findings indicate that m6A RNA methylation regulates cardiac homeostasis and hypertrophy. However, the molecular mechanisms of YTHDF proteins involved in HF progression are still unclear.

In this study, we explored the possible roles of YTHDF proteins during HF progression using human failing hearts samples and mice model of TAC. We found that YTHDF2 mRNA and protein level, but not YTHDF1 or YTHDF3, were significantly increased in HF tissues and cardiomyocytes with hypertrophic stimulation. YTHDF2 overexpression could efficiently alleviate cardiac hypertrophy. Furthermore, YTHDF2 protein interacts with Myh7 (beta-myosin heavy chain) mRNA, an important cardiac hypertrophy marker, to induce Myh7 mRNA decoy in an m6A-dependent manner, thereby inhibiting HF development. Our study highlights the functional importance of YTHDF2-dependent cardiac m6A mRNA regulation during heart failure, and provides a novel mechanistic insight into the therapeutic mechanisms of YTHDF2.

## Results

### YTHDF2 protein expression increases in human heart failure samples

**Fig. 1:**
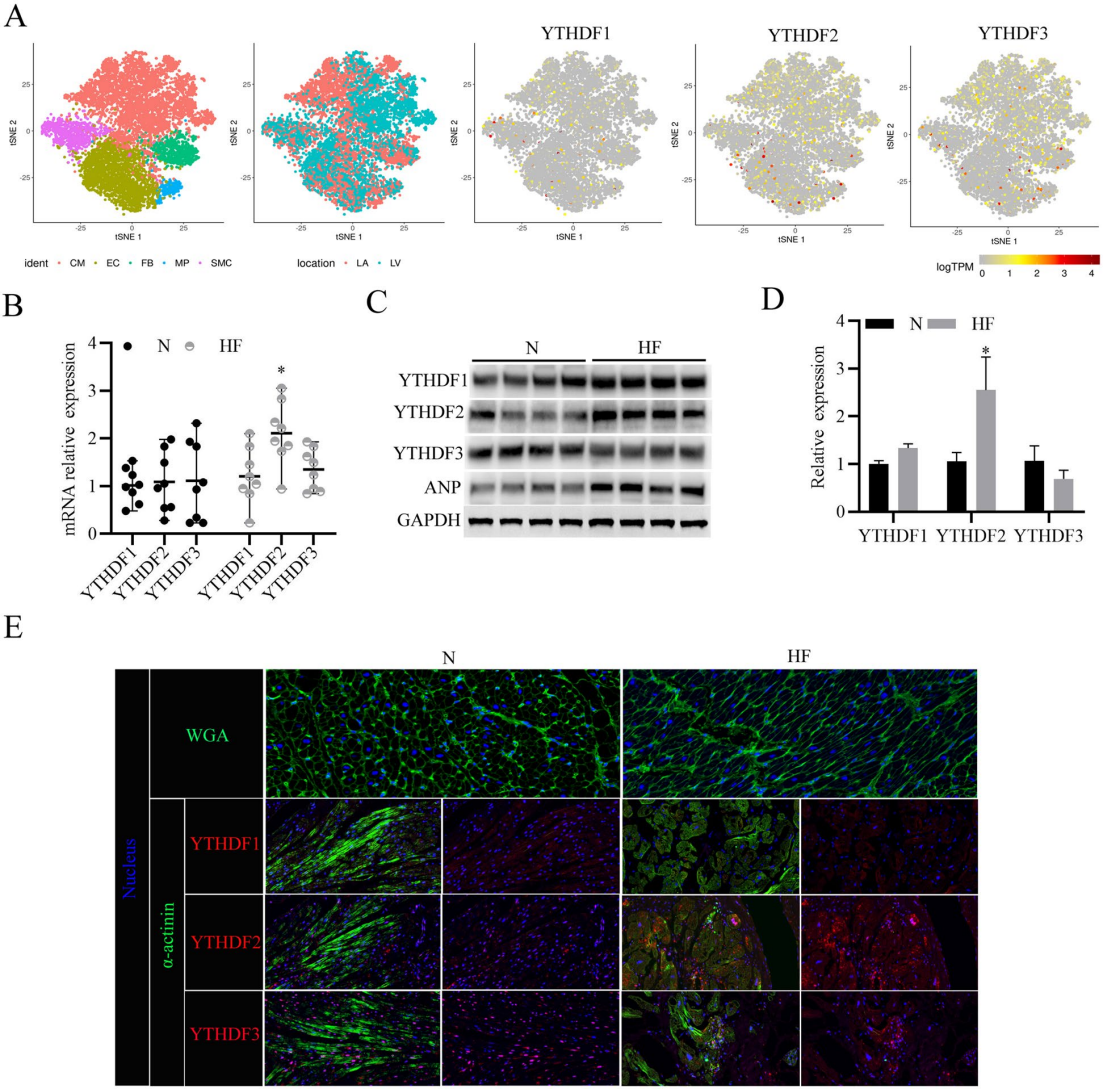
YTHDF2 protein expression increases in human heart failure samples. **A** 2D visualization of single-cell clusters of TAC-model mouse heart tissues by tSNE. (*CM* cardiomyocytes; *EC* endothelial cells, *FB* fibroblasts, *MP* macrophages, *SMC* smooth muscle cells); 2D visualization of single-cell clusters present in the left ventricle (LV) and the left atrial (LA); 2D visualization of YTHDF1/2/3 gene expressions in the single-cell clusters present in the left ventricle. **B** RT-PCR analysis of YTHDF1/2/3 mRNA expressions in the clinical HF (n = 8) tissues and the normal (N) heart tissues (n = 8). **C** Western blotting analysis of YTHDF1/2/3 protein expressions in the clinical HF (n = 4) tissues and the normal (N) heart tissues (n = 4). ANP was used as a positive control, and GAPDH was used as a loading control. **D** Densitometry quantification of protein expressions. **E** WGA staining (Green) was used to measure cardiomyocyte size of the clinical HF tissues and the normal (N) heart tissues; Immunofluorescence analysis of YTHDF1/2/3 (Red) expressions in cardiomyocytes (stained with anti-α-actinin antibodies, Green). Nucleus was stained with DAPI (Blue). ^*^P < 0.05

Recent studies find that methylase Mettl3 and demethylase FTO function essential roles during HF progression through regulating m6A methylation level [16, 17]. To explore the roles of YTHDF family proteins during HF, we analyzed recently published single-cell RNA-seq data of TAC-model mouse heart tissue (GSE120064) [17], and found 5 major cellular clusters, including cardiomyocytes (CM), endothelial cells (EC), fibroblasts (FB), macrophages (MP), and smooth muscle cells (SMC) distributed in the HF tissue (Fig. 1A). Besides, more cardiomyocytes distributed in the left ventricle (LV) than in the left atrial (LA) (Fig. 1A). Furthermore, we found YTHDF1/2/3 proteins are distributed in all of the 5 major cellular clusters (Fig. 1A). Subsequently, we analyzed the expression levels of YTHDF1/2/3 in the clinical normal and HF tissues, and found that YTHDF2 mRNA levels (Fig. 1B) and protein levels (Fig. 1C, D) in the HF tissues were significantly higher than those in the normal heart tissues. As expected, the protein expression levels of Atrial Natriuretic Peptide (ANP) were significantly increased in the HF tissues, which is consistent with previous study [18] (Fig. 1C). Moreover, the wheat germ agglutinin (WGA) staining of myocardial tissue showed that myocardial cells in the HF tissues were larger than those the normal heart tissues, and immunofluorescent staining further confirmed that YTHDF2 protein expressions were notably increased in the HF tissues (Fig. 1E). Collectively, these results suggest that YTHDF2 protein increases in human HF samples.

### YTHDF2 protein expression increases in the cell and animal model of cardiac hypertrophy

**Fig. 2:**
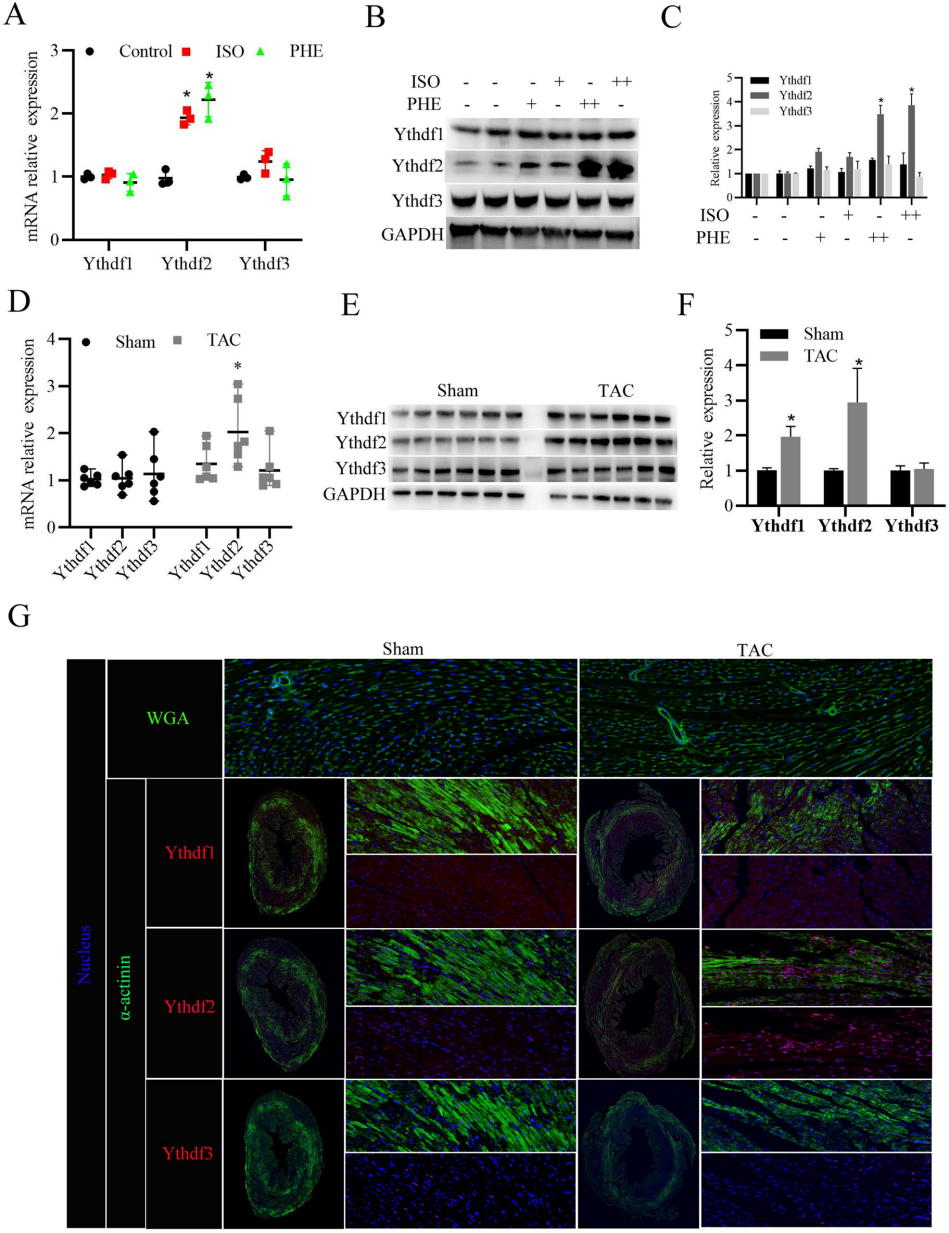
YTHDF2 protein expression increases in the heart tissues of mice with cardiac hypertrophy. **A** RT-PCR analysis of YTHDF1/2/3 mRNA expressions in the primary cardiomyocytes stimulated with isoproterenol (ISO, 10 μmol/l), phenylephrine (PHE, 50 μmol/l), or DMSO (as control) for 24 h. **B** Western blotting analysis of YTHDF1/2/3 protein expressions in the primary cardiomyocytes stimulated with ISO (+ , 10 μmol/l; +  + , 20 μmol/l), PHE (+ , 50 μmol/l; +  + , 80 μmol/l), or DMSO (as control) for 24 h. **C** Densitometry quantification of protein expressions. **D** RT-PCR analysis of YTHDF1/2/3 mRNA expressions in the heart tissues of mice after 4 weeks of TAC surgery (n = 6) or Sham surgery (n = 6). **E** Western blotting analysis of YTHDF1/2/3 protein expressions in the heart tissues of Sham (n = 6) or TAC (n = 6) mice. **F** Densitometry quantification of protein expressions. **G** WGA staining (Green) was used to measure cardiomyocyte size in the heart tissues of Sham or TAC mice; Immunofluorescence analysis of YTHDF1/2/3 (Red) expressions in cardiomyocytes (stained with anti-α-actinin antibodies, Green). Nucleus was stained with DAPI (Blue). ^*^P < 0.05

Subsequently, we further investigated the expression levels of YTHDF1/2/3 in the primary cardiomyocytes treated with ISO or PHE to induce myocardial hypertrophy in vitro, and in the mice with cardiac hypertrophy induced by TAC in vivo. As shown in Fig. 2A–C, YTHDF2 mRNA and protein expression level was significantly increased in either ISO or PHE treated cardiomyocytes, compared to control. Whereas, ISO or PHE stimulation did not obviously affect YTHDF1 and YTHDF3 mRNA and protein expression. Furthermore, consistent with the in *vitro* results, YTHDF2 mRNA and protein expression level was also significantly increased in the mice with cardiac hypertrophy induced by TAC (Fig. 2D–F). Interestingly, YTHDF1 protein, but not mRNA expression level also significantly upregulated in the mice with cardiac hypertrophy induced by TAC. Moreover, immunofluorescent staining further confirmed that YTHDF2 protein expressions were notably increased in the hypertrophic mice hearts (Fig. 2G). Collectively, these results suggest that YTHDF2 was highly expressed in the hypertrophic mice hearts and ISO or PHE-induced cardiomyocytes.

### YTHDF2 plays a protective role on cardiac hypertrophy in vivo

**Fig. 3:**
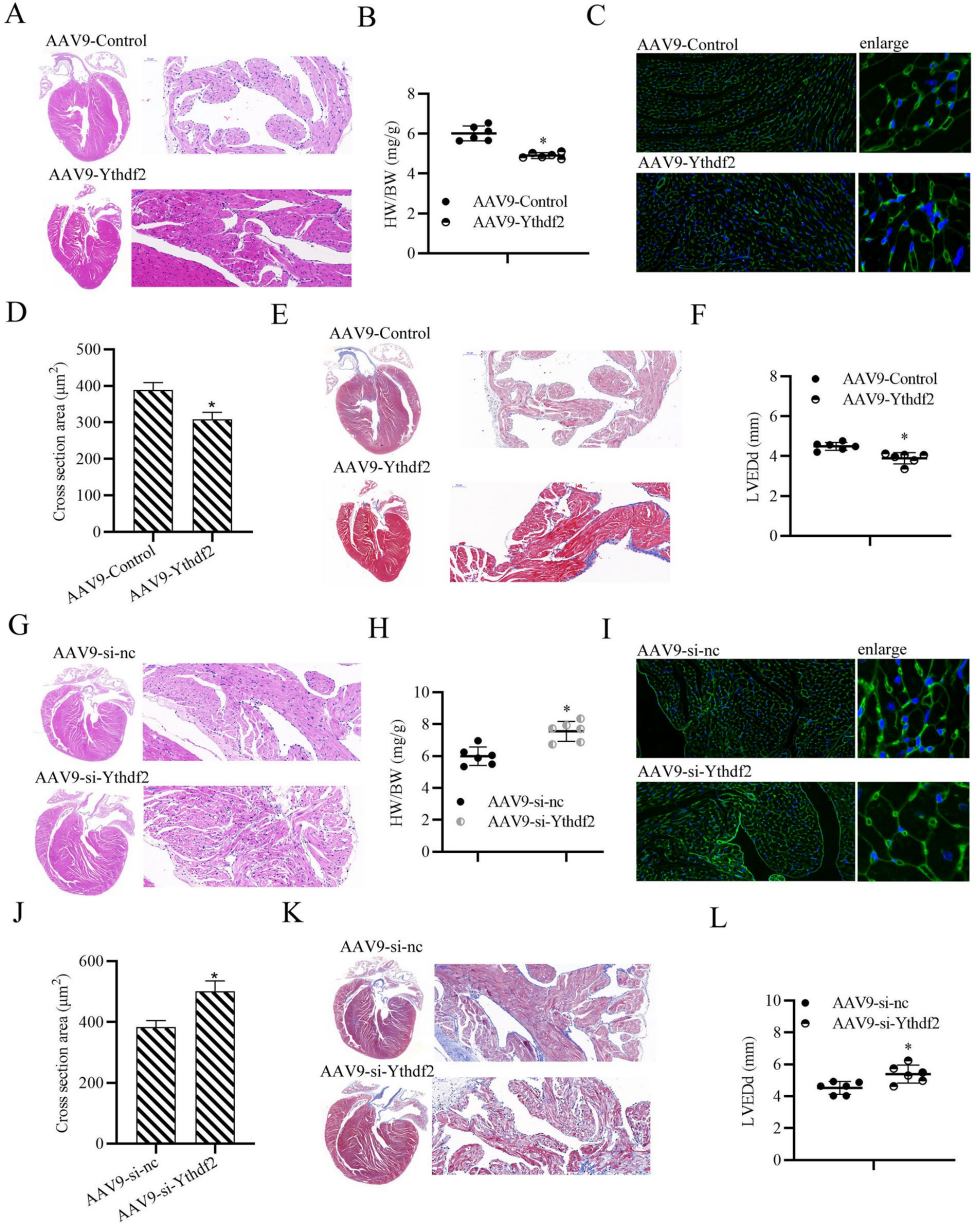
YTHDF2 plays a protective role on cardiac hypertrophy in vivo. **A** HE staining for the assessment of gross cardiac enlargement of mice injected with AAV-YTHDF2 or control AAV through tail vein for 3 weeks, then subjected to TAC for 4 weeks. **B** Statistical analysis of the heart weight (HW)/body weight (BW) ratios of mice subjected to TAC surgery for 4 weeks (n = 6 mice per group; *p < 0.05 versus sham group). **C** WGA staining (Green) was used to measure cardiomyocyte size in the heart tissues of TAC mice. **D** Statistical analysis of the cross section areas of the indicated group. **E** Representative results of the Masson staining to assess fibrosis of the mouse heart tissues. **F** Detection of left ventricular end-diastolic diameter (LVEDd) in mice, n = 6. **G** HE staining for the assessment of gross cardiac enlargement of mice injected with AAV-si-YTHDF2 or control AAV through tail vein for 3 weeks, then subjected to TAC for 4 weeks. **H** Statistical analysis of the heart weight (HW)/body weight (BW) ratios of mice subjected to TAC surgery for 4 weeks (n = 6 mice per group; *p < 0.05 versus sham group). **I** WGA staining (Green) was used to measure cardiomyocyte size in the heart tissues of TAC mice. **J** Statistical analysis of the cross section areas of the insdicated group. **K** Representative results of the Masson staining to assess fibrosis of the mouse heart tissues. **L** Detection of left ventricular end-diastolic diameter (LVEDd) in mice, n = 6. ^*^P < 0.05

To further explore the specific role of YTHDF2 during cardiac hypertrophy development, gain/loss-of-function experiments were performed. The mice were injected with AAV9 viral particles carrying YTHDF2 (AAV-YTHDF2) through tail intravenous injection, or control AAV for 3 weeks, and the mice were later subjected to TAC to induce cardiac hypertrophy. As shown in Fig. 3A–D, YTHDF2 overexpressing mice showed a smaller heart size (Fig. 3A), lower HW/BW (Fig. 3B), and fewer enlarged cardiomyocytes (Fig. 3C, D), compared with the control mice after 4 weeks of TAC surgery. Consistently, YTHDF2 overexpressing mice also showed less fibrosis detected by the Masson staining (Fig. 3E), and lower LVEDd measured by echocardiographic evaluation (Fig. 3F). Moreover, as expected, YTHDF2-knockdown mice exhibited a larger heart size (Fig. 3G), higher HW/BW (Fig. 3H), more enlarged cardiomyocytes (Fig. 3I, J), more fibrosis (Fig. 3K), and higher LVEDd (Fig. 3L). Collectively, these results indicate that YTHDF2 overexpression alleviates cardiac hypertrophy in vivo.

### Functional analysis of YTHDF2-interacting proteins in cardiomyocytes stimulated with ISO

**Fig. 4:**
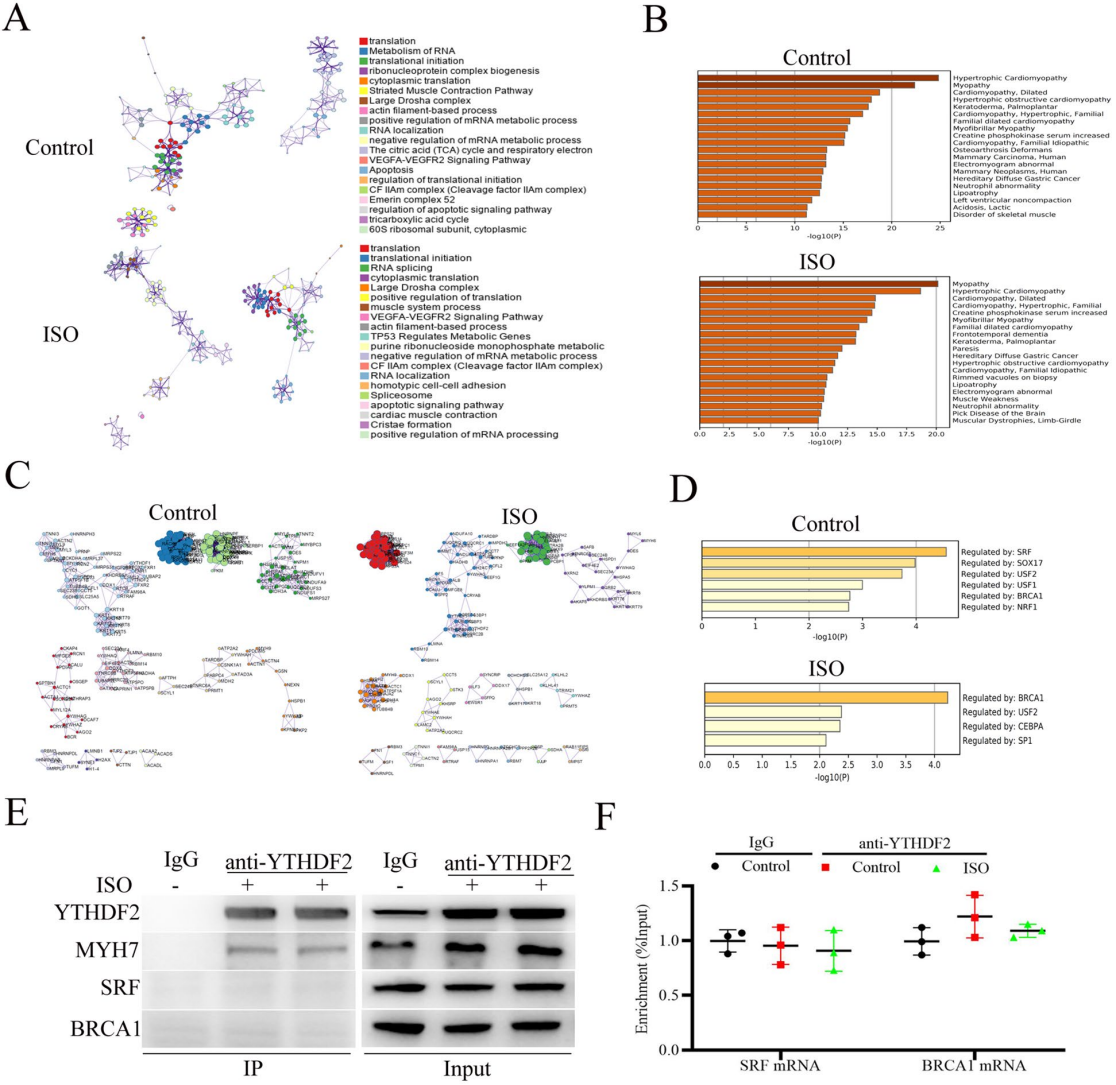
Functional analysis of YTHDF2-interacting proteins in cardiomyocytes stimulated with ISO. (A and B) GO (**A**) and KEGG (**B**) annotation enrichment analysis of the YTHDF2 interacting proteins identified by mass spectrometry using Metascape (https://metascape.org). **C** Protein–protein interaction (PPI) network analysis of the YTHDF2 interacting proteins by Cytoscape. **D** Transcriptional factor analysis of the YTHDF2 interacting proteins. **E** Co-IP analysis of the interaction between YTHDF2 and MYH7/SRF/BRCA1 in the cardiomyocytes stimulated with ISO (10 μmol/l) or DMSO (as control). **F** RIP analysis of the interaction between YTHDF2 and SRF/BRCA1 mRNA in the cardiomyocytes stimulated with ISO (10 μmol/l) or DMSO (as control). ^*^P < 0.05

To further investigate the molecular mechanism of YTHDF2 alleviating cardiac hypertrophy, YTHDF2-interacting proteins in the cardiomyocytes treated with DMSO (as control) or ISO were immunoprecipitated and then identified by mass spectrometry. As shown in Fig. 4A, B, GO and KEGG annotation enrichment analyses showed that ISO stimulation did not significantly affect the molecular functions or associated with diseases of YTHDF2 interacting proteins. Besides, protein–protein interaction (PPI) network analysis showed that ISO stimulation did not significantly affect the interactions between YTHDF2 and the proteins identified by mass spectrometry (Fig. 4C). Moreover, transcriptional regulation analysis showed that ISO stimulation did not obviously affect the transcription of the genes coding proteins potentially interacted with YTHDF2 (Fig. 4D). Among the identified proteins by mass spectrometry, MYH7 (beta-myosin heavy chain) has been reported as an important cardiac hypertrophy marker [19]. Thus, we firstly hypothesized whether ISO stimulation affects the interaction between YTHDF2 and MYH7. Co-IP analysis showed that YTHDF2 indeed interacted with MYH7 in cardiomyocytes. But, ISO stimulation did not affect the interaction of YTHDF2 and MYH7 (Fig. 4E), suggesting YTHDF2 may be not affect cardiac hypertrophy by interacting with MYH7. In addition, RNA immunoprecipitation (RIP) analysis showed that ISO stimulation did not affect YTHDF2 interacted with SRF (serum response factor) mRNA or BRCA1 (Breast carcinoma 1) mRNA (Fig. 4F).

### YTHDF2 alleviating myocardial hypertrophy depends on interacting with Myh7 mRNA

**Fig. 5:**
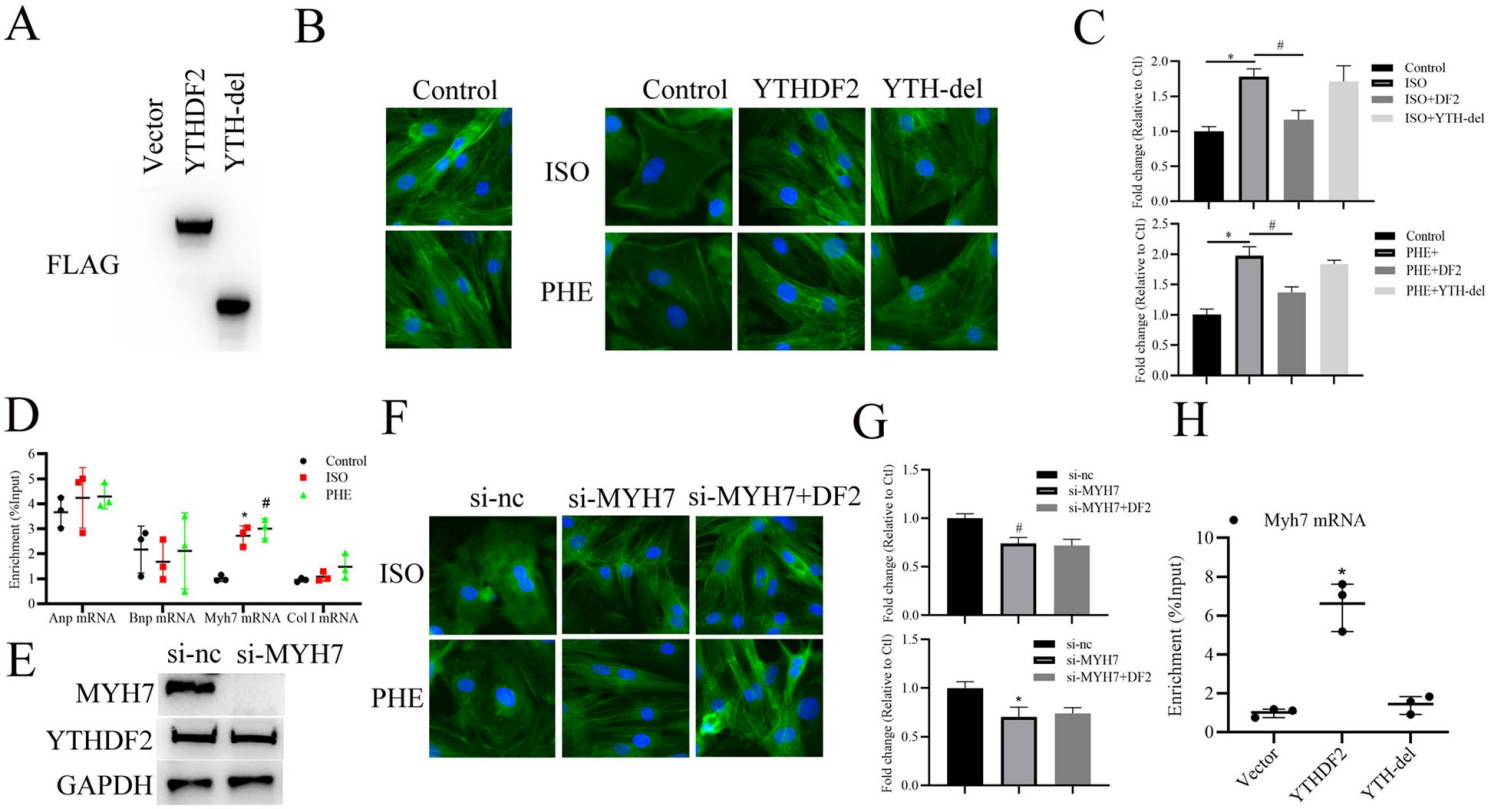
YTHDF2 alleviating myocardial hypertrophy dependents on interacting with Myh7 mRNA. **A** Western blotting analysis of YTHDF2 and its mutant expression in the primary cardiomyocytes infected with the indicated FLAG-tagged AAV vectors. **B** Microscopic images of the CMs. Cells were seeded in 24-well plates, infected with the indicated FLAG-tagged AAV vectors for 48 h, then treated with ISO, PHE or not for 48 h. Both cell groups were then stained with antibodies directed against α-actinin (Green) and with DAPI (Blue) for nuclear staining. **C** Cell size was measured in 10 fields/well in all groups (n = 3 independent experiments; n > 50 cells per experimental group; *p < 0.05). **D** RIP analysis of the interaction between YTHDF2 and ANP/BNP/Myh7/Collagen type 1 (col 1) mRNA in the cardiomyocytes stimulated with ISO, PHE or DMSO. **E** Western blotting analysis of MYH7 and YTHDF2 expression in the primary cardiomyocytes transfected with si-nc or si-Myh7. **F** Microscopic images of the CMs. Cells were seeded in 24-well plates, transfected with si-nc or si-Myh7 and AAV9-FLAG-YTHDF2 vector for 48 h, then treated with ISO or PHE for 48 h. Both cell groups were then stained with antibodies directed against α-actinin (Green) and with DAPI (Blue) for nuclear staining. **G** Cell size was measured in 10 fields/well in all groups. (n = 3 independent experiments; n > 50 cells per experimental group; *p < 0.05). **H** RIP analysis of YTHDF2 or YTH-del binding on the Myh7 mRNA (n = 3 independent experiments, *p < 0.05)

Next, we further explored whether the RNA binding ability of YTHDF2 is involved in YTHDF2 alleviating cardiac hypertrophy. Plasmid expressing YTH domain-delated YTHDF2 (YTH-del), which is responsible for YTHDF2’s RNA binding ability [20], was synthesized. As shown in Fig. 5A–C, consistent with the results of Fig. 3C, overexpression of YTHDF2 evidently suppressed ISO or PHE-induced myocardial hypertrophy, whereas abolishment of YTHDF2’s RNA binding ability partially reversed the effect of overexpression of YTHDF2, suggesting that YTHDF2-mediated RNA metabolism is involved in the progression of cardiac hypertrophy. Furthermore, we detected whether YTHDF2 interacts with the mRNAs of cardiac hypertrophy makers, including ANP (atrial Natriuretic Peptide), BNP (brain natriuretic peptide), and MYH7 by RIP. The results showed that ISO or PHE stimulation evidently increased the interaction of YTHDF2 and Myh7 mRNA, but not Anp or Bnp mRNA (Fig. 5D). Furthermore, we found that knockdown of MYH7 significantly inhibited ISO or PHE-induced myocardial hypertrophy, and overexpression of YTHDF2 did not further alleviate myocardial hypertrophy (Fig. 5E–G). In addtion, RIP analysis showed that YTHDF2 directly binds to Myh7 mRNA, but deletion of the YTH domain of YTHDF2 cancels this binding (Fig. 5H). Collectively, these results indicate that YTHDF2 interacting with Myh7 mRNA via YTH domain to alleviate cardiac hypertrophy.

### YTHDF2 recognizes the m6A site on Myh7 mRNA to promote its degradation

**Fig. 6:**
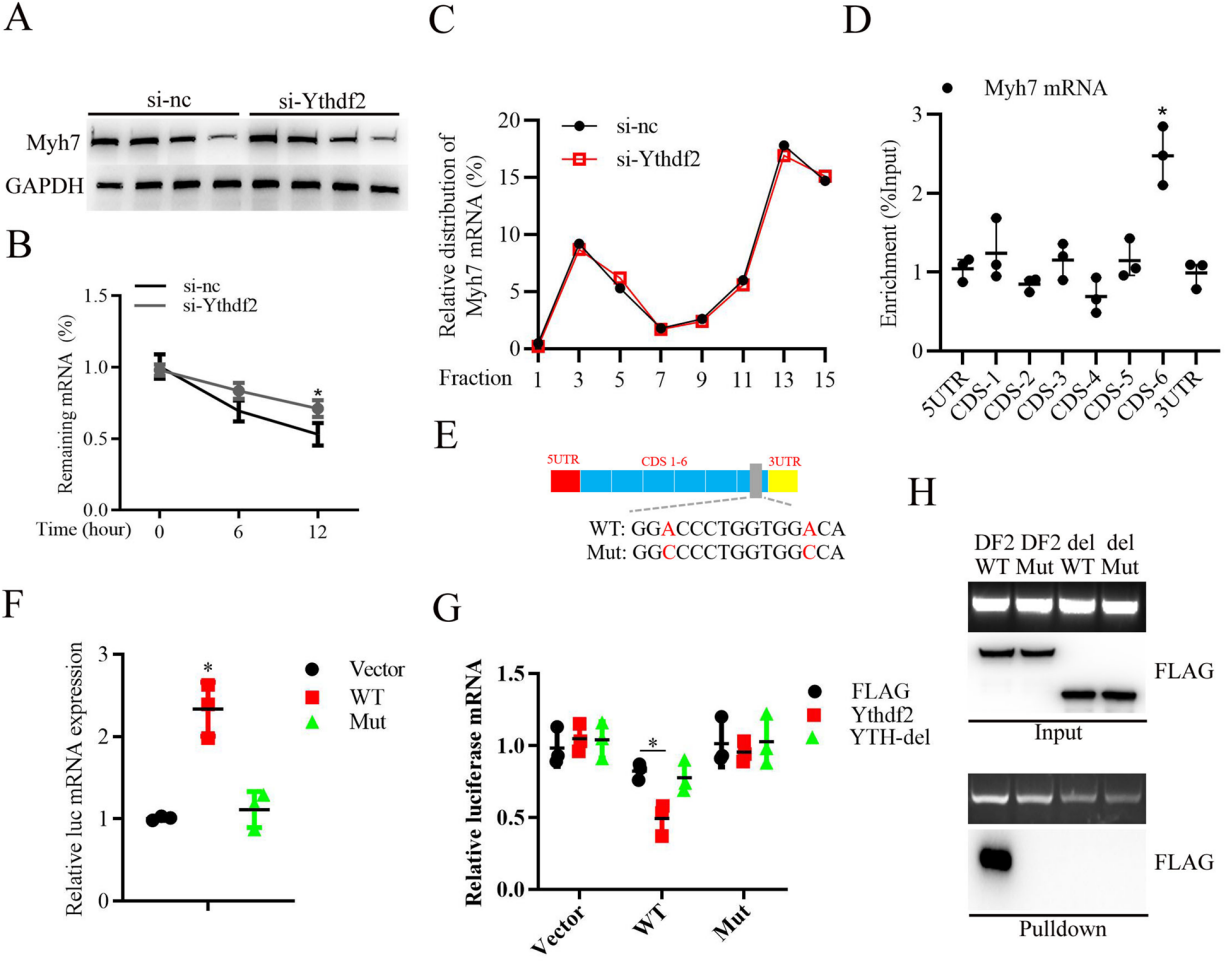
YTHDF2 recognizes the m6A site on Myh7 mRNA to promote its degradation. **A** Western blotting analysis of MYH7 expression in the primary cardiomyocytes transfected with si-nc or si-YTHDF2 for 24 h, then treated with 50 μg/ml CHX for the indicated times. **B** RT-PCR analysis of Myh7 mRNA expression in the primary cardiomyocytes transfected with si-NC or si-YTHDF2 for 24 h, then treated with ActD treatment (10 μg/ml) for the indicated times. **C** Amount of Myh7 mRNA in various polysome fractions was analyzed by RT-PCR. **D** RIP analysis of the m6A modification on the indicated regions of Myh7 mRNA (5’UTR, 5’Untranslated Region; 3’UTR, 3’Untranslated Region; CDS, Coding sequences). **E** Schematic diagram of the potential m6A modification sites on Myh7 mRNA. **F** RIP analysis of the m6A modification on the wild type (WT) or mutant (Mut) Myh7 mRNA CDS-6 region, which was cloned into pGL3-luciferase reporter plasmid. **G** Cells were co-transfected with pCMV3-C-FLAG-YTHDF2 (YTHDF2), pCMV3-C-FLAG-YTHDF2 mutant (YTH del), or pCMV3-C-FLAG vector (Flag), and pGL3-luciferase-wild type (WT) Myh7 mRNA CDS-6 region, pGL3-luciferase-mutant (Mut) Myh7 mRNA CDS-6 region, or pGL3-luciferase vector (Vector). RIPs were performed to detect the association between YTHDF2 or YTH-del and WT or Mut Myh7 mRNA CDS-6 region. **H** RNA pulldown analysis of the interaction between biotinylated-Myh7 mRNA CDS-6 (WT) or mutant Myh7 mRNA CDS-6 (Mut) and pCMV3-C-FLAG-YTHDF2 (DF2) or pCMV3-C-FLAG-YTHDF2 mutant (del). ^*^P < 0.05

Given that YTHDF2 interacting with Myh7 mRNA in cardiomyocytes (Fig. 5D), we further explored whether YTHDF2 affected MYH7 expression. As shown in Fig. 6A, B, knockdown of YTHDF2 did not affect MYH7 protein stability (Fig. 6A) analyzed by protein stability assay, whereas knockdown of YTHDF2 significantly increased Myh7 mRNA stability (Fig. 6B). Furthermore, YTHDF2 knockdown did not affect the distribution of Myh7 mRNA in the polysome (F13-F14) or subpolysome fractions (F3-F4) (Fig. 6C), indicating that YTHDF2 did not affect Myh7 mRNA translation. Considering that m6A modification plays important roles in regulating mRNA decoy [21], we further whether m6A modification occurs on Myh7 mRNA. RIP analysis with anti-m6A antibodies showed that m6A modification occurs in the CDS (coding sequence)-6 region of Myh7 mRNA (Fig. 6D), and two potential m6A modification-motif were identified in the region (Fig. 6E). Subsequently, pGL3-luciferase reporter plasmid coding the wide type (WT) or mutant (Mut) sequences of CDS-6 region of Myh7 mRNA was transfected into Hela cell lines. Then, RIP analysis showed that WT, but not Mut plasmid was specifically immunoprecipitated with anti-m6A antibodies (Fig. 6F). Moreover, YTH domain-delated YTHDF2 (YTH-del) significantly canceled the binding of WT Myh7 CDS-6 but not Mut (Fig. 6G). In addition, RNA pulldown analysis with biotinylated-Myh7 mRNA CDS-6 (WT) or mutant Myh7 mRNA CDS-6 (Mut) probe showed that WT Myh7 mRNA CDS-6 specifically interacted with YTHDF2 protein (Fig. 6H). Overall, these results indicate that YTHDF2 recognizes the m6A site on Myh7 mRNA to promote its degradation.

### ISO or PHE stimulation promotes YTHDF2 protein expression through enhancing Ythdf2 mRNA stability in cardiomyocytes

**Fig. 7:**
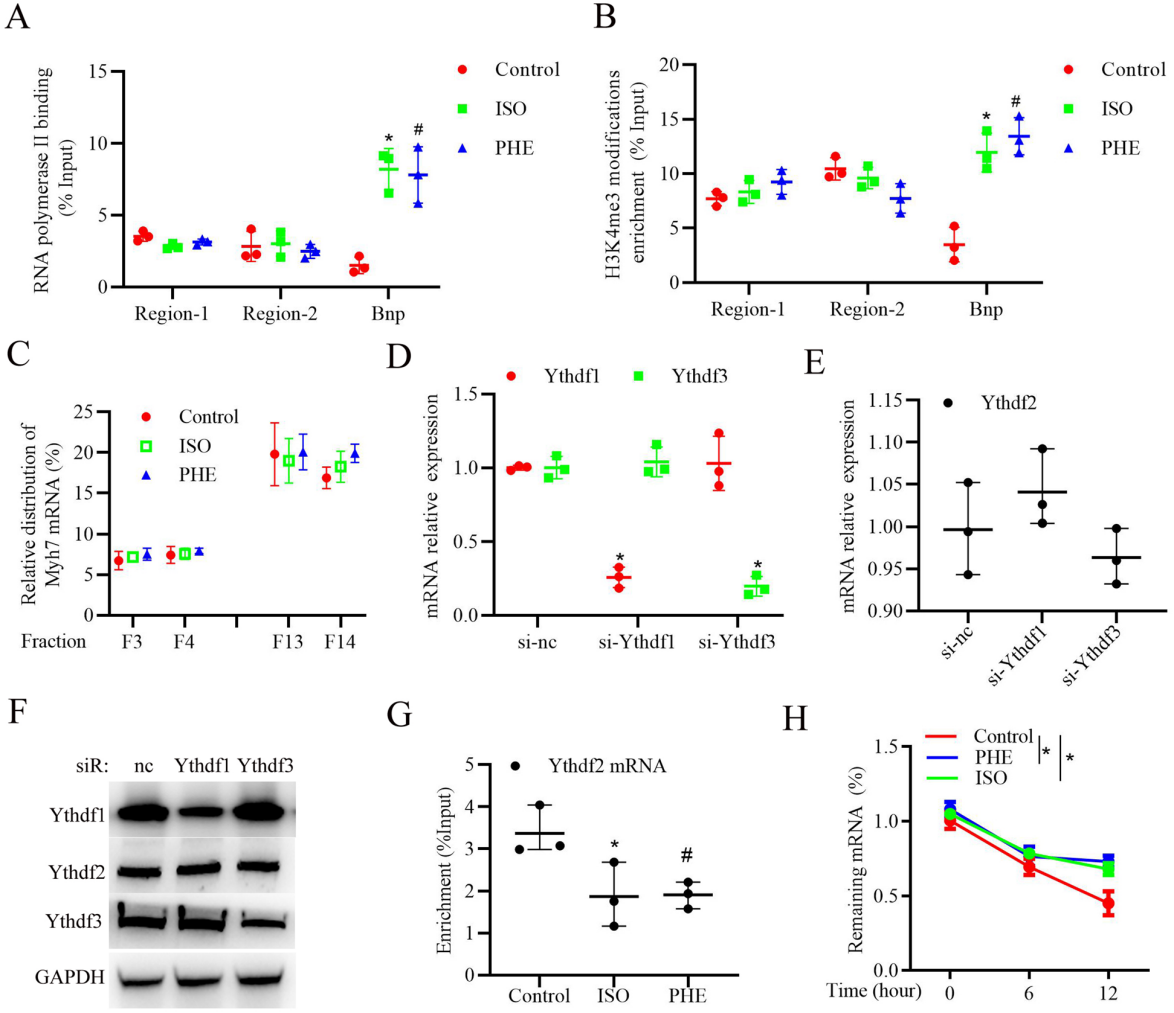
ISO or PHE stimulation promotes YTHDF2 protein expression through enhancing Ythdf2 mRNA stability in cardiomyocytes. **A** CHIP analysis of the interaction between RNA polymerase II and YTHDF2 gene promoter region 1/2 or BNP gene promoter in cardiomyocytes stimulated with ISO, PHE or DMSO (as control). **B** CHIP analysis of H3K4me3 modification on the YTHDF2 gene promoter region 1/2 or BNP gene promoter in cardiomyocytes stimulated with ISO, PHE or DMSO (as control). **C** RT-PCR analysis of the amount of YTHDF2 mRNA in various polysome fractions in cardiomyocytes stimulated with ISO, PHE or DMSO (as control). **D** RT-PCR analysis of YTHDF1/3 mRNA expressions in cardiomyocytes transfected with si-nc, si-YTHDF1, or si-YTHDF3. **E** RT-PCR analysis of YTHDF2 mRNA expressions in cardiomyocytes transfected with si-nc, si-YTHDF1, or si-YTHDF3. **F** Western blotting analysis of YTHDF1/2/3 protein expressions in cardiomyocytes transfected with si-nc, si-YTHDF1, or si-YTHDF3. **G** RIP analysis of the interaction between YTHDF2 protein and YTHDF2 mRNA in cardiomyocytes stimulated with ISO, PHE or DMSO (as control). **H** RT-PCR analysis of YTHDF2 mRNA expression in the primary cardiomyocytes treated with ActD treatment (10 μg/ml) for the indicated times. ^*^P < 0.05

To further explore the underlying mechanism of YTHDF2 upregulation during HF progression, we firstly examined the interaction between RNA polymerase II, which is mainly responsible for transcriptional initiation of gene [22], and Ythdf2 gene promoter regions [range from 0 to −2000 bp from transcription start site, including region-1 (0 to − 1000 bp) and region-2 (− 1001 bp to 2000 bp)]. The result of CHIP with antibodies against RNA polymerase II showed that ISO or PHE stimulation did not significantly affect Ythdf2 gene transcription in cardiomyocytes, but evidently promoted Bnp gene transcription (Fig. 7A). Recently, Histone H3 lysine 4 (H3K4) trimethylation (H3K4me3), generally correlates with transcriptional activation of genes [23], has been reported to play essential roles in cardiac homeostasis [24]. We further examined the effects of ISO or PHE on the H3K4me3 modification of Ythdf2 gene, and CHIP analysis showed that ISO or PHE stimulation did not significantly affect H3K4me3 modification of Ythdf2 gene (Fig. 7B), but significantly promoted H3K4me3 modification on Bnp gene. Besides, ISO or PHE stimulation also did not affect the distribution of Ythdf2 mRNA in the polysome fraction (F13-F14) or subpolysome fractions (F3-F4) [25, 26] (Fig. 7C). Furthermore, we detected the effects of YTHDF1/3 proteins on Ythdf2 expression, and found that knockdown of YTHDF1 or YTHDF3 did not affect Ythdf2 mRNA or protein expressions (Fig. 7D–F). Unexpected, RIP analysis showed that ISO or PHE stimulation significantly decreased YTHDF2 protein interacting with Ythdf2 mRNA (Fig. 7G), suggesting YTHDF2 may have a self-regulation. Moreover, ISO or PHE stimulation evidently suppressed Ythdf2 mRNA degradation in cardiomyocytes (Fig. 7H), indicating that YTHDF2 protein could regulate Ythdf2 mRNA stability under ISO or PHE stimulation. Taken together, these results indicate that ISO or PHE stimulation promotes YTHDF2 self-regulation and protein upregulation through enhancing Ythdf2 mRNA stability in cardiomyocytes.

## Discussion

HF, which leads to most of cardiovascular hospitalizations and death all over the world, still lacks therapeutic targets and effective drugs nowadays [27]. Remodeling of the m6A landscape plays an important role in the development of cardiomyocyte hypertrophy and HF [28]. m6A content has been reported to be increased in human heart failure samples, and increased expression of the m6A RNA methylase METTL3 was sufficient to promote cardiomyocyte hypertrophy both in vitro and in vivo [28]. Consistently, upregulation of m6A RNA demethylase FTO expression in failing mouse hearts improves cardiac contractile function [29]. In this study, we for the first time explored the roles of the m6A “Reader” YTHDF family during HF development, and found that YTHDF2, but not YTHDF1 or YTHDF3, increases in human HF samples, mice HF samples, and cardiomyocytes with hypertrophic stimulation. Furthermore, we found that YTHDF2 suppresses cardiac hypertrophy via Myh7 mRNA decoy in an m6A-dependent manner. Our study highlights the functional importance of m6A “Reader” proteins-dependent cardiac m6A mRNA regulation during HF, and provides a novel mechanistic insight into the therapeutic mechanisms of YTHDF2.

YTHDF family proteins, including YTHDF1, YTHDF2 and YTHDF3, positively or negatively regulate RNA metabolism through recognizing m6A modification via YTH domain. YTHDF1 is reported to promote the translation of m6A mRNA by enhancing the ribosome assembly of m6A mRNA and interacting with the initiation factor [12]. YTHDF2 mainly regulates the stability of target mRNA through transforming the m6A RNA from the translation state to the degradation state [30]. YTHDF3 can promote the translation and degradation of mRNA [31]. In this study, we find that YTHDF2 specifically interacts with Myh7 mRNA, an important cardiac hypertrophy marker, to induce Myh7 mRNA decoy in an m6a-dependent manner in cardiomyocytes, and hypertrophic stimulation further enhances the interaction of YTHDF2 and Myh7 mRNA. To date, more than 400 hypertrophic cardiomyopathy-associated mutations have been described in Myh7 gene [32], and patients with likely pathogenic or pathogenic variation in Myh7 gene have a higher rate of incident atrial fibrillation independent of clinical and echocardiographic factors [33]. Whether m6A modification is associated with Myh7 gene mutation needs more attention in the following studies. Besides, we also find that YTHDF2 interacts with MYH7 protein in cardiomyocytes, whereas hypertrophic stimulation does not affect the interaction of YTHDF2 and MYH7 protein. Whether YTHDF2 interacting with MYH7 protein influences myocardial active contraction, such as isometric force levels, shortening velocity, and calcium sensitivity of force generation, needs to be further studied. In addition, even if no significant differences of YTHDF1/3 expression between the normal heart tissues and HF tissues, the detailed roles of YTHDF1/3 during HF development still require deeper investigation.

As an m6A reader protein, YTHDF2 participates in various biological processes, including migration, invasion, metastasis, proliferation, apoptosis, cell cycle, cell viability, cell adhesion, differentiation and inflammation, in many diseases [34–36]. Through immunoprecipitation accompanied with mass spectrometry analysis, we find that the proteins-interacted with YTHDF2 in cardiomyocytes are mainly related to hypertrophic cardiomyopathy, myopathy and dilated cardiomyopathy, suggesting YTHDF2 may function essential roles on cardiac homeostasis. Among the identified proteins, MYH6 (α-myosin heavy chain) and MYH9 play important roles in heart development, and are associated with HF [37, 38]. In addition, Protein arginine methyltransferase 5 (PRMT5), a protein arginine methyltransferase that catalyzes the symmetrical dimethylation of arginine residues within target proteins, is an important regulator of myocardial hypertrophic signaling [39]. Besides regulating mRNA decoy, whether the protective effect of YTHDF2 on cardiac hypertrophy and HF also related to these proteins requires further investigation in the following studies.

In this study, we find that YTHDF2 suppresses cardiac hypertrophy through regulating Myh7 mRNA decoy in vitro using the primary mice cardiomyocytes stimulated with ISO or PHE, and in vivo using TAC mouse model. It is reported that the Myh isoforms are differentially expressed in the ventricular myocardium of rodents and humans [40–45]. The Myh composition of the ventricular myocardium of rodents is major α-Myh (Myh6) [40–42], whereas that of humans is major β-Myh (Myh7) [43–45]. Thus, hiPSC (human induced pluripotent derived stem cell)-differentiated cardiomyocytes should be used to further uncover the regulatory mechanism of YTHDF2 on human cardiac hypertrophy. In addition, although the differential Myh composition of the ventricular myocardium of rodents and humans, the protein level alteration of Myh (down-regulation of α-Myh and up-regulation of β-Myh) was consistent in both human HF and experimental animal HF models [46]. In rats, α-Myh protein could decrease from 93–97% to 22–71%, while β-Myh protein could increase from 3–6% to 29–78% during heart failure [46]. For the humans, α-Myh mRNA was expressed at considerable levels (30% of total MyHC mRNA) in the non-failing human left ventricles and was substantially decreased (reduced to 2%) in end-stage failing human ventricles [47]. The Myh isoform switching is a major contributing factor to the decline in cross-bridge kinetics observed in experimental rodent HF models [49]. Whether YTHDF2 is also involved in regulating the Myh isoform switching during human HF progression needs to be investigated in the following studies.

In conclusion, out study explores the role of YTHDF family proteins during HF development, and indicates YTHDF2 suppresses cardiac hypertrophy and HF via Myh7 mRNA decoy in an m6A-dependent manner. This study highlights the functional importance of YTHDF2-dependent cardiac m6A mRNA regulation during heart failure, and sheds light on potential new targets for hypertrophy and HF therapy.

## Methods and materials

### Single-cell RNA sequencing (scRNA-seq) analysis

Single cell RNA sequence data from heart tissues of TAC-model mice was obtained from the Gene Expression Omnibus (GEO) database (GSE120064) [17]. Cells expressing < 200 or > 7000 genes in normal heart samples, and < 200 or > 5000 genes in heart failure samples were filtered out for exclusion of noncell or cell aggregates. The data were log‐normalized and highly variable genes were selected using the FindVariableFeatures function and downstream procedures were performed using the ScaleData and runPCA function. Then, FindClusters function was performed to cluster cells and uniform manifold approximation and projection (UMAP) with R package Seurat was used to visualize clusters, as described in the vignettes (https://satijalab.org/seurat/vignettes.html).

### Patient samples

Human left ventricular samples were collected and used in this study, including control samples (nonfailing, nontransplantable hearts, n = 8), and heart failure samples (end-stage dilated cardiomyopathy, n = 8). All heart failure patients were diagnosed with dilated cardiomyopathy with EF < 25% (systolic heart failure). All heart failure patients were diagnosed with heart failure (Function Capacity IV, Objective Assessment D, based on New York Heart Association functional classifcation) at least 3 months before heart transplantation. Written informed consent was obtained from patients. Samples were collected in accordance with human research protocol approved by the Research Ethics Committee of the First Affiliated Hospital, College of Medicine, Zhejiang University.

### RNA extraction and real-time qPCR analysis

The total RNA of heart tissues and isolated mice primary cardiomyocytes was extracted with TriZol reagent (Invitrogen, USA), and then reverse-transcribed into cDNA using PrimeScript RT Master Mix (Takara, Japan). SYBR Premix Ex TaqII (Takara, Japan) kit was used for qRT-PCR detection with ABI PRISM 7500 Detection System (ABI, USA). Values were normalized by GAPDH. The primers are listed in Additional file 1: Table S1.

### Western blotting

Proteins were extracted from heart tissues or isolated mice primary cardiomyocytes with RIPA Lysis Buffer (Beyotime, China), and protein concentration was determined using the BCA protein assay kit (Beyotime, China). Protein samples were separated on a 10% SDS-PAGE gel and then transferred to PVDF membranes (Millipore, USA). The membranes were blocked with 5% non-fat milk for 1 h at room temperature and then incubated overnight at 4 °C with the following primary antibodies: YTHDF1 (ab220162, Abcam, USA), YTHDF2 (ab246514, Abcam, USA), YTHDF3 (ab220161, Abcam, USA), ANP (ab262703, Abcam, USA), flag (ab205606, Abcam, USA), and GAPDH (ab8245, Abcam, USA), followed by incubation with secondary antibodies for 1 h. The protein blots were visualized using the ECL kit (Pierce, USA).

### Transverse aortic constriction (TAC) mouse model

All animal experimental procedures were approved by the Animal Care Ethics Committee of the first affiliated hospital Zhejiang university school of medicine, and the experiments were performed in compliance with the “Guide for the Care and Use of Laboratory Animals” from the US National Institute of Health. Eight-week-old male C57BL/6 J mice were purchased from Model Animal Research Center of Nanjing University, and housed in the Laboratory Animals Center of the first affiliated hospital Zhejiang university school of medicine, with controlled temperature and humidity. HF was induced in mice by transverse aortic constriction (TAC), as previously described [50]. Briefly, mice were anesthetized with 0.3% sodium pentobarbital (75 mg·kg^−1^) intraperitoneally, and the aortic arch was tied with a 6–0 nylon suture between the brachiocephalic and left common artery with a homemade L-shaped 26G cushion needle. After ligation, the needle was quickly removed, and the skin was closed. The sham operation was identical, except that the thread was not ligated. Moreover, mice were injected with rAAV9 (4 × 10^11^ vector genomes (vg)/mouse) carrying an empty vector, YTHDF2 or YTH-del via the tail vein.

### Cell isolation and culture

Primary cardiomyocytes were isolated from adult mice (male, 8 weeks old) or neonatal mice (1–2 days). Briefly, after wash, hearts were cut up in saline with HEPES-buffer. Then, the tissues were decentralized and incubated in HEPES-buffered saline solution with 0.14 mg/ml collagenase and 1.2 mg/ml pancreatin at 37 °C. The cells collected by centrifugation were re-suspended in Dulbecco’s modified Eagle medium/F-12 with 0.1 mM ascorbate, 5% heat-inactivated fetal bovine serum (FBS), 100 U/ml penicillin, insulin-transferring-sodium selenite media supplement, 0.1 mM bromodeoxyuridine and 100 μg/ml streptomycin. After pre-plated at 37 °C for 1 h, the cells were plated in dishes coated by 10 μg/ml laminin. For inducing hypertrophy, cells were treated with treated with isoproterenol (ISO, 10 μmol/l, Sigma, USA) or phenylephrine (PHE, 50 μmol/l, Sigma, USA) for 24 h.

### Echocardiographic evaluation

4 weeks after TAC surgery, the cardiac function of mice by echocardiography using a 30-MHz high-frequency scanhead (VisualSonics Vevo770, VisualSonics, Canada). End-systole and end-diastole were defined as the phases in which the smallest and largest areas of the left ventricular (LV), respectively, were obtained. LV end-diastolic diameter (LVEDd) were measured from the LV M-mode at the mid-papillary muscle level. After echocardiographic evaluation, mice were euthanized by cervical dislocation 4 weeks post-operatively. Mice hearts were dissected and weighed or measured to compare the heart weight (HW)/body weight (BW) ratios.

### Histological study

The heart tissue sections were paraffin embedded and cut into 5-μM serial sections and then were stained with a hematoxylin and eosin (HE) staining kit (Byotime, China) to assess myocardial pathological changes, and a Masson staining kit (SbjBio, China) was used to evaluate cardiac fibrosis, respectively, according to the manufacture’s instruction.

### Immunofluorescence

The heart tissue sections were blocked with goat serum at room temperature for 30 min, and then stained with fluorescein conjugated wheat germ agglutinin staining (Alexa Fluor‐488, Invitrogen, CA) was used to evaluate cardiomyocyte size. Myocyte nucleus was stained using 4′, 6‐diamidino‐2‐phenylindole (DAPI, Sigma, USA). For each group, approximately 50–100 randomly chosen cardiomyocytes were analyzed by using Image J software to measure the cross-sectional cardiomyocyte area. To evaluate the expressions of YTHDF1/2/3 proteins in heart tissue, heart tissue sections were blocked, incubated with primary antibodies against α-actinin (Sigma, USA) and YTHDF1/2/3 (Abcam, USA) at 4 °C overnight, and treated with fluorescence-conjugated secondary antibody for 1 h at 37 °C. The nuclei were counterstained with DAPI. After washing, the sections were imaged by a Zeiss Confocal Microscope Imaging System (Carl Zeiss, Germany).

### Co-immunoprecipitation (Co-IP) assay and mass spectrometry analysis

Primary cardiomyocytes were treated with ISO or DMSO (as control) for 24 h. Then, the cells were lysed with Cell lysis buffer for Western and IP (Beyotime, China), and centrifuged to collect the supernatant. One tenth of the supernatant was retained for the input immunoblot, while the rest (300 μg proteins) was incubated with anti-YTHDF2 or rabbit IgG at 4 °C overnight, followed by further incubation with 10 μl protein A/G-agarose beads (Cell Signaling Technology, USA) for another 4 h. The bound proteins were subjected to washing three times for a total of 30 min and then eluted by boiling for 5 min in the loading buffer. Immunocomplexes were subjected into SDS-PAGE electrophoresis and the gel was then stained with the Fast Silver Stain Kit (Beyotime, Shanghai, China). Then, the gel was analyzed independently by reverse-phase liquid chromatography coupled with tandem mass spectrometry using ACQUITYTM UPLC-QTOF analysis platform at Beijing Protein Innovation (Beijing, China). For detecting the interaction between YTHDF2 and MYH7/SRF/BRCA1, the immunoprecipitated proteins were resolved by SDS-PAGE and detected by Western blotting with anti-bodies against MYH7 (22,280–1-AP, ProteinTech, China), SRF (ab252868, Abcam, USA), or BRCA1 (ab238983, Abcam, USA).

### Bioinformatic analysis

Metascape (http://metascape.org/gp/index.html#/main/step1) was applied to analyze the Gene ontology (GO) functional, disease association and transcription factors annotation of the proteins immunoprecipitated with YTHDF2 in primary cardiomyocytes treated with ISO or DMSO (as control).

### RNA‐binding protein immunoprecipitation assay (RIP assay)

Magna RIP Kit (Millipore, USA) was used to perform the RIP assay. In brief, Primary cardiomyocytes were treated with ISO or DMSO (as control) for 24 h, and then lysed with RIP lysis buffer. The supernatant was incubated with antibodies against YTHDF2 antibody (Abcam, USA) overnight at 4 °C. Then, 50 μl A/G magnetic beads were added to the supernatant and incubated for 6 h. After immobilizing the magnetic bead bound complexes with a magnetic separator (Millipore USA), supernatants were used to extract RNA with PCA (phenol: chloroform: isoamyl alcohol) reagent at a ratio of 125:24:1 (Aladdin, USA). The results were quantified by RT-PCR.

### siRNAs transfection

siRNA against mouse Ythdf2 mRNA, and siRNA against mouse Myh7 mRNA were synthesized by GnenPharma (Shanghai, China). Plasmids or siRNAs were transiently transfected into cardiomyocytes using jetprime transfection reagents (Polyplus, France) according to the manufacturer’s instructions.

### Protein and mRNA stability assays

Primary cardiomyocytes were transfected with si-NC or si-Ythdf2 for 24 h, then treated with 50 μg/ml cycloheximide (CHX; Sigma Aldrich) to block protein synthesis, or 5 mg/ml of Actinomycin D (ActD; Sigma Aldrich) to block RNA transcription. After culturing at the various time points, cells were collected for Western blotting, or subjected to RNA extraction. RT-PCR were used to analyze the levels of MYH7 mRNA.

### Polysome profiling

Primary cardiomyocytes were transfected with si-NC or si-Ythdf2 for 24 h, then treated with 100 μg/ml of cycloheximide for 15 min. Then cells are lysed by polysome buffer [200 mmol/L KCl, 15 mmol/L MgCl2, 1% Triton X100, 100 μg/mL cycloheximide, 20 mmol/L heparin, and 100 U/mL RNase Inhibitor (Takara), 1X cocktail] for 15 min on ice, lysates were centrifuged (14,000 rpm for 15 min), and the supernatant was layered onto a 5 to 50% sucrose gradient. Gradients were then centrifuged at 38,000 rpm for 130 min at 4 °C and polysome-bound fractions were collected using an ISCO Density Gradient Fractionation System (ISCO, Lincoln, NE) with continuous monitoring based on A260nm wavelength. The RNA in each fraction was extracted using Trizol reagent (Invitrogen) and analyzed by RT-PCR.

### Luciferase assay

pGL3-basic luciferase reporter vector containing the wild-type and mutant Myh7 mRNA CDS-6 sequences (NCBI Reference Sequence: NM_001361607.1) was synthesized by GnenPharma (Shanghai, China), respectively. 293 T cells were transfected with 750 ng of pGL3-basic luciferase reporter vector (Vector), wild-type pGL3-Myh7 mRNA CDS-6 (WT) or mutant pGL3-Myh7 mRNA CDS-6 (Mut) in combination with pCMV3-C-FLAG vector, pCMV3-C-FLAG-YTHDF2 plasmid, or pCMV3-C-FLAG-YTHDF2 YTH-del plasmid using lipo2000 transfection reagent (Life Technologies, USA). At 24 h after transfection, we lysed the cells and measured the luciferase activity by luciferase reporter assay system (Beyotime Biotechnology, Shanghai, China). All of the experiments were performed in triplicate.

### Biotin-coupled probe RNA pull down assay

Biotinylated-Myh7 mRNA CDS-6 (WT) or mutant Myh7 mRNA CDS-6 (Mut) probe was synthesized by RiboBio (Guangzhou, China). Plasmids or siRNAs were transiently transfected into cardiomyocytes using jetprime DNA transfection reagents (Polyplus, France) according to the manufacturer’s instructions. Cardiomyocytes were transfected with pCMV3-C-FLAG vector, pCMV3-C-FLAG-YTHDF2 plasmid, or pCMV3-C-FLAG-YTHDF2 YTH-del plasmid using jetprime DNA transfection reagents (Polyplus, France). After transfection for 48 h, the cells were collected and lysed. The lysate was then incubated with biotin-labeled probe. Next, the biotin-coupled RNA complex was pulled down using streptavidin-coated magnetic beads adsorption. The enriched YTHDF2 was analyzed by western blot analysis.

### Chromatin immunoprecipitation (ChIP)

Chip assay was performed with the simple ChIP Enzymatic Chromatin IP Kit (Magnetic Beads) (Cell Signaling Technologies, USA), according to the manufacturer's instructions. Briefly, 5 × 10^6^ cardiomyocytes were treated with ISO, PHE, or DMSO (as control) for 24 h. Then, cells were cross‐linked with 37% formaldehyde (final concentration of 1%) and incubated for 10 min at room temperature. The chromatin fraction was digested with micrococcal nuclease and sonicated to obtain the desired fragment length of 150–900 bp. The protein‐DNA complexes were immunoprecipitated using anti-RNA polymerase II (abcam, ab252854), or H3K4me3 (abcam, ab213224) antibodies. Then, the immunoprecipitated DNA was amplified by RT-PCR. The primers used are listed in Additional file 1: Table S1. The signals obtained from each immunoprecipitation reaction were expressed as a percent of the total input chromatin with the following equation:

$$Enrichment\,percentage = 2\%  \times 2CT\,input\,samples - CTIP\,samples$$

### Statistical analysis

All statistical analysis was performed by GraphPad Prism 7.0. Data were expressed as mean ± standard deviation (SD). Differences between two groups were determined by using unpaired Student’s t-test. Furthermore, differences among multiple groups were determined by using ANOVA. Statistical significance was considered when P < 0.05.

## Supplementary Information

**Additional file 1: Table S1.** Primers used in this study.

## Data Availability

All data generated and/or analyzed during this study are included in this published article.
