# Supplementary material for: YTHDF2 alleviates cardiac hypertrophy via regulating Myh7 mRNA decoy
Source: Cell Biosci. 2021 Jul 15;11:132. doi: 10.1186/s13578-021-00649-7 (PMC8281596; doi:10.1186/s13578-021-00649-7)
Supplement: Supplementary file 1 — Additional file 1: Table S1. Primers used in this study. [file 13578_2021_649_MOESM1_ESM.docx]

**Table S1. Primers used in this study.**

| **Genes** | **Primers for RT-CR** | | | **Primer Sequenses (5’-3’)** |
| --- | --- | --- | --- | --- |
| **Human** | |  |  | |
| YTHDF1 | | Forward | ACCTGTCCAGCTATTACCCG | |
| YTHDF1 | | Reverse | TGGTGAGGTATGGAATCGGAG | |
| YTHDF2 | | Forward | AGCCCCACTTCCTACCAGATG | |
| YTHDF2 | | Reverse | TGAGAACTGTTATTTCCCCATGC | |
| YTHDF3 | | Forward | TCAGAGTAACAGCTATCCACCA | |
| YTHDF3 | | Reverse | GGTTGTCAGATATGGCATAGGCT | |
| GAPDH | | Forward | GGAGCGAGATCCCTCCAAAAT | |
| GAPDH | | Reverse | GGCTGTTGTCATACTTCTCATGG | |
| **Mouse** | |  |  | |
| YTHDF1 | | Forward | ACAGTTACCCCTCGATGAGTG | |
| YTHDF1 | | Reverse | GGTAGTGAGATACGGGATGGGA | |
| YTHDF2 | | Forward | GAGCAGAGACCAAAAGGTCAAG | |
| YTHDF2 | | Reverse | CTGTGGGCTCAAGTAAGGTTC | |
| YTHDF3 | | Forward | GATCAGCCTATGCCATATCTGAC | |
| YTHDF3 | | Reverse | CCCCTGGTTGACTAAAAACACC | |
| GAPDH | | Forward | AGGTCGGTGTGAACGGATTTG | |
| GAPDH | | Reverse | TGTAGACCATGTAGTTGAGGTCA | |
| Myh7 | | Forward | ACTGTCAACACTAAGAGGGTCA | |
| Myh7 | | Reverse | TTGGATGATTTGATCTTCCAGGG | |
| **Genes** | | **Primers for RIP** | **Primer Sequenses (5’-3’)** | |
| SRF | | Forward | GGCCGCGTGAAGATCAAGAT | |
| SRF | | Reverse | CACATGGCCTGTCTCACTGG | |
| BRCA1 | | Forward | CGAATCTGAGTCCCCTAAAGAGC | |
| BRCA1 | | Reverse | AAGCAACTTGACCTTGGGGTA | |
| ANP | | Forward | GCTTCCAGGCCATATTGGAG | |
| ANP | | Reverse | GGGGGCATGACCTCATCTT | |
| BNP | | Forward | GAGGTCACTCCTATCCTCTGG | |
| BNP | | Reverse | GCCATTTCCTCCGACTTTTCTC | |
| Col Ⅰ | | Forward | GTAACTTCGTGCCTAGCAACA | |
| Col Ⅰ | | Reverse | CCTTTGTCAGAATACTGAGCAGC | |
| Myh7 5’UTR | | Forward | CAGGTCTGGCTCTGAGCATT | |
| Myh7 5’UTR | | Reverse | GGCTGAGCCTTGGATTCTCA | |
| Myh7 CDS-1 | | Forward | CCTGCGGAAGTCTGAGAAGG | |
| Myh7 CDS-1 | | Reverse | CCCAATGGCGGCAATAACAG | |
| Myh7 CDS-2 | | Forward | GAACCAGACGGCACTGAAGA | |
| Myh7 CDS-2 | | Reverse | CAGGTGGTTGTCGTACAGCT | |
| Myh7 CDS-3 | | Forward | CTCCAGGGGTGATGGACAAC | |
| Myh7 CDS-3 | | Reverse | TTGACTCGCCCAAACTCCTC | |
| Myh7 CDS-4 | | Forward | ACCTGACAGAGGAGATGGCT | |
| Myh7 CDS-4 | | Reverse | CCTCCTCCAGCCTTTCACTG | |
| Myh7 CDS-5 | | Forward | ATGAATGAGCACCGGAGCAA | |
| Myh7 CDS-5 | | Reverse | CCTCTGCTTCTTGTCCAGGG | |
| Myh7 CDS-6 | | Forward | GGATGCAGACCTATCCCAGC | |
| Myh7 CDS-6 | | Reverse | TTGCGGAACTTGGACAGGTT | |
| Myh7 3’UTR | | Forward | CTCTTGTGCTACCCAGCT | |
| Myh7 3’UTR | | Reverse | TTTTGGCTTTAAGGAA | |
| **Genes** | | **siRNA** | **Sequenses (5’-3’)** | |
|  | | Negative control | UUCUCCGAACGUGUCACGUTT | |
|  | | YTHDF2 | AAGGACGTTCCCAATAGCCAA | |
| **Genes** | | **Primers for CHIP** | **Primer Sequenses (5’-3’)** | |
| YTHDF2 promoter region-1 | | Forward | TTTGGTGGTGCATGCCTTTG | |
| YTHDF2 promoter region-1 | | Reverse | CTCACCCGTATGCACAGTGT | |
| YTHDF2 promoter region-2 | | Forward | CCCACAGCATACCCACCATT | |
| YTHDF2 promoter region-2 | | Reverse | GTCAACCTCATGGCTCAGCT | |
| BNP promoter | | Forward | GAGAGAGAGAGAGAGAGAGG | |
| BNP promoter | | Reverse | GTTGGTCAAGGACAATGACC | |
